# Identification of antibodies against phospholipase A2 receptor peptides in PLA2R-associated membranous nephropathy with negative circulating anti-PLA2R antibodies

**DOI:** 10.3389/fimmu.2026.1816719

**Published:** 2026-05-28

**Authors:** Miao Wang, Liu Chen, Bing-jia Yan, Jin-ying Wang, Lei Liu, Zhao Cui, Ming-hui Zhao

**Affiliations:** 1 Renal Division, Peking University First Hospital; Institute of Nephrology, Peking University; Key Laboratory of Renal Disease, Ministry of Health of China; Key Laboratory of CKD Prevention and Treatment, Ministry of Education of China;, Beijing, China; 2 Key Laboratory of Bioorganic Phosphorus Chemistry and Chemical Biology (Ministry of Education), Department of Chemistry, Tsinghua University, Beijing, China; 3 Peking-Tsinghua Center for Life Sciences, Beijing, China

**Keywords:** epitope mapping, linear peptides, membranous nephropathy, PLA2R, seronegative

## Abstract

**Background:**

About 30% of patients with phospholipase A2 receptor (PLA2R) associated membranous nephropathy (MN) have negative circulating anti-PLA2R antibodies. We investigated whether these patients harbor antibodies against linear PLA2R peptides, with the aim of improving serological detection.

**Methods:**

We synthesized 123 sequential overlapping linear peptides spanning the extracellular region of PLA2R across ten domains. Circulating IgG antibodies to these peptides were measured by ELISA in 42 biopsy-proven PLA2R-associated MN patients with negative circulating anti-PLA2R antibodies.

**Results:**

Twenty-six of 42 (61.9%) anti-PLA2R-negative MN patients reacted with at least one PLA2R peptide. Overall, 68 of 123 (55.3%) peptides were recognized, with the highest recognition frequencies for CysR-11 (21.4%), CTLD7-1 (14.3%) and CTLD7-2 (16.7%). Reactivity to these peptides was rare in patients with minimal change disease, focal segmental glomerulosclerosis, IgA nephropathy, hepatitis B virus-associated MN, or tumor-associated MN. Combined any positivity of antibodies to the three peptides yielded a sensitivity of 43.7% and a specificity of 81.3% for identifying PLA2R-associated MN in the absence of circulating anti-PLA2R antibodies. In inhibition assays, none of the three peptides detectably blocked binding of circulating anti-PLA2R antibodies to full-length PLA2R under the experimental conditions used. Among 32 anti-PLA2R-positive MN patients, 28 (87.5%) also reacted with PLA2R peptides, with frequent recognition of CysR-5, CTLD1-7, CTLD4-7, CTLD5-6, CTLD5-9, CTLD6-2, and CTLD7-2.

**Conclusions:**

Antibodies to linear PLA2R peptides are detectable in a substantial proportion of PLA2R-associated MN patients who are seronegative by conventional anti-PLA2R assays. CysR-11, CTLD7-1, and CTLD7–2 may serve as exploratory candidate biomarkers, and their pathogenic relevance warrants further study.

## Introduction

Primary membranous nephropathy (MN) is a leading cause of adult-onset nephrotic syndrome and is now recognized as an organ-specific autoimmune glomerular disease characterized by subepithelial immune deposits. M-type phospholipase A2 receptor (PLA2R), expressed on podocytes, is the major autoantigen in most cases, and glomerular PLA2R colocalizes with IgG4 in deposits (1). Although PLA2R has been confirmed as the major antigen of MN, a significant number of patients remain antibody-negative, with over 30% of MN patients testing negative in serum assays according to a systematic review meta-analysis (2). Anti-PLA2R antibodies are widely used for diagnosis and for assessing immunologic activity, treatment response, and prognosis (3–6). However, in seronegative PLA2R-associated MN, kidney biopsy remains the main diagnostic tool and there is no established circulating biomarker for reflecting autoimmune activity during treatment and follow-up.

The extracellular region of PLA2R comprises an N-terminal cysteine-rich region (CysR), a fibronectin type II domain (FnII), and eight C-type lectin-like domains (CTLD) (7, 8). In patients with detectable anti-PLA2R antibodies, several antigenic epitopes have been described, including both linear sequences and conformational epitopes within CysR, CTLD1, and CTLD7 (9–12). Epitope spreading of the anti-PLA2R response has been observed during disease progression and is associates with poor prognosis (11). Notably, sera from patients who are negative in full-length PLA2R assays may fail to recognize the CysR-FnII-CTLD1 fragment (9). In other autoimmune kidney diseases, such as anti-glomerular basement membrane (GBM) disease and ANCA-associated vasculitis, antibodies to linear peptides can precede or accompany antibodies to conformational epitopes through intramolecular epitope spreading; peptide-specific antibodies and antigen-specific CD4+ T cells can also contribute to kidney injury (13–20).

Therefore, we hypothesized that patients with PLA2R-associated MN who are seronegative for anti-PLA2R antibodies may nonetheless harbor antibodies to linear PLA2R peptides. We synthesized 123 overlapping peptides (15–20 amino acids) spanning the extracellular region of PLA2R and screened 42 biopsy-proven seronegative PLA2R-associated MN patients for peptide-reactive IgG. Defining such epitopes may provide insight into the immunologic features of seronegative PLA2R-associated MN and identify exploratory candidate serological markers that could complement, but not replace, established biopsy-based diagnostic approaches.

## Materials and methods

### Patients

Forty-two patients with biopsy-proven PLA2R-associated MN and negative circulating anti-PLA2R antibodies were enrolled from 2015 to 2019. All patients underwent kidney biopsy and showed granular IgG and C3 deposition and strong PLA2R staining along glomerular capillary walls, diffuse thickening of the glomerular basement membrane (GBM), and subepithelial electron-dense deposits. Circulating anti-PLA2R antibodies were measured by enzyme-linked immunosorbent assay (ELISA) using purified PLA2R as the solid-phase antigen (Euroimmun, Luebeck, Germany), and all results were negative (<14 U/mL).

Secondary causes of MN were evaluated by review of clinical history, physical examination, laboratory testing, and biopsy findings. Systemic lupus erythematosus and other systemic autoimmune diseases were excluded based on the absence of suggestive clinical manifestations and routine serological evaluation, including antinuclear antibody, anti-double-stranded DNA antibody, serum complement C3 and C4 levels, and other autoimmune tests when clinically indicated (21). Viral hepatitis was assessed by hepatitis B virus and hepatitis C virus serology, and HBV-associated MN was defined by evidence of viral replication. Malignancy-associated MN was evaluated according to clinical history, age-appropriate screening, imaging, and tumor markers when indicated. Patients with drug exposure or heavy metal exposure potentially associated with secondary MN were excluded.

In this study, PLA2R-associated MN was defined by positive glomerular PLA2R staining in kidney biopsy specimens, regardless of circulating anti-PLA2R antibody status, after exclusion of clinically evident secondary causes at baseline. Therefore, the seronegative cohort should be interpreted as a selected group of PLA2R-staining-positive MN patients rather than an unselected population of all patients with seronegative MN.

We also included 32 MN patients with positive circulating anti-PLA2R antibodies. In addition, six patients with HBV-associated MN (with viral replication) and three patients with tumor-associated MN were included; all were negative for circulating anti-PLA2R antibodies. Disease controls included 31 patients with primary focal segmental glomerulosclerosis (FSGS), 20 with minimal change disease (MCD), and 20 with IgA nephropathy. Plasma from 40 healthy donors served as healthy controls.

All blood samples were collected on the day of kidney biopsy, before initiation of steroids or other immunosuppressive agents. Clinical data were extracted from medical records at diagnosis and during follow-up. Peptide antibody testing was performed as part of this exploratory research study and was not used to guide clinical management. Treatment decisions were made according to routine clinical parameters, including proteinuria, serum albumin, kidney function, pathological findings, and physician judgment. Therefore, peptide reactivity did not influence medication choice in this cohort.

The study complied with the Declaration of Helsinki and was approved by the Ethics Committee of Peking University First Hospital. Written informed consent was obtained for collection of tissue and blood samples.

### Preparation of PLA2R linear peptides and protein

The complete sequence of the extracellular domain of human PLA2R1 isoform 1 (accession no. NP_031392.3; amino acids 1-1397) was subcloned into the pcDNA3.1 vector for expression in HEK293 cells. Cells were cultured at 37 °C in 5% CO2, and culture supernatant was collected over 5 days after transfection (22). Recombinant protein was purified via the HA tag and verified by SDS-PAGE and Western blot.

A panel of 123 sequential overlapping peptides was synthesized to cover the extracellular region of PLA2R. Each peptide was 15–20 amino acids long, with 8–10 amino acids of overlap between adjacent peptides, and carried an N-terminal biotin. Peptides were synthesized using an automatic peptide synthesizer with 9-fluorenylmethoxycarbonyl (Fmoc) chemistry (Beijing Scilight Biotechnology Co., Beijing, China) and purified by preparative reverse-phase C18 HPLC. Purity and identity were confirmed by analytical HPLC and mass spectrometry, and peptides with purity >98% were used.

### Determination of antigen specificity by ELISA

Streptavidin (5 μg/mL) in coating buffer (0.05 M bicarbonate buffer, pH 9.6) was used to coat polystyrene microtiter plates overnight at 4 °C. Biotinylated peptides (10 μg/mL) were added to half of the wells and incubated at 37 °C for 60 minutes; the remaining wells were coated with buffer alone to serve as antigen-free controls. After blocking with 1% bovine serum albumin (BSA) at 37 °C for 60 minutes, plasma samples were diluted 1:100 in PBS containing 0.1% Tween-20 (PBST) and incubated at 37 °C for 60 minutes. Bound IgG was detected with alkaline phosphatase-conjugated goat anti-human IgG (Fc-specific; Sigma, St. Louis, MO, USA) at 1:5000. p-Nitrophenyl phosphate (1 mg/mL; Sigma) in substrate buffer (1 M diethanolamine and 0.5 mM magnesium chloride, pH 9.8) was used for color development, and absorbance was read at 405 nm (Bio-Rad, Tokyo, Japan) after 30 minutes. Each plate included positive, negative, and blank controls. All plasma samples were tested in duplicate wells for each peptide. For each sample, the OD value from the corresponding antigen-free well was subtracted to correct for non-specific background binding, and the mean OD value of duplicate wells was used for subsequent analysis. The intra-assay and inter-assay coefficients of variation for the peptide ELISAs were all below 15%. Cut-off values for each peptide were defined as the mean + 2 SD (23) of OD values from 40 healthy donors.

### Determination of cross-reactivity by inhibition ELISA

PLA2R antigen (6 μg/mL) in coating buffer (0.05 M bicarbonate buffer, pH 9.6) was used to coat half of the wells and incubated at 37 °C for 60 minutes; the remaining wells were coated with buffer alone. After blocking with 1% BSA at 37 °C for 60 minutes, plasma samples were diluted 1:100 in PBST and preincubated with linear peptides (0, 1, 2.5, 5, 10, 15, or 20 μg/mL) at 37 °C for 60 minutes. Binding to coated PLA2R was detected using alkaline phosphatase-conjugated goat anti-human IgG (Fc-specific; Sigma) at 1:5000 and p-nitrophenyl phosphate substrate as described above. Absorbance at 405 nm was measured after 30 minutes.

### Detection of PLA2R expression in kidneys

For immunohistochemistry, paraffin sections of kidney biopsy specimens were dewaxed in xylene and rehydrated through graded ethanol. Antigen retrieval was performed in citrate buffer by microwave heating (high power for 5 minutes followed by low power for 9 minutes), then slides were cooled to room temperature and washed with PBS. Endogenous peroxidase activity was quenched with freshly prepared 3% hydrogen peroxide for 10 minutes. Non-specific binding was blocked with 3% BSA at 37 °C for 60 minutes. Rabbit anti-human PLA2R antibody (HPA012657; Sigma) was applied at 1:1000 and incubated at 4 °C overnight; blank controls were incubated with 3% BSA without primary antibody. A rabbit two-step detection kit (PV9001, ZSGB-Bio, Beijing, China) was used according to the manufacturer’s instructions. Slides were counterstained with hematoxylin, dehydrated, and mounted. Staining was assessed semiquantitatively at ×200 magnification.

### Detection of THSD7A expression in kidneys

THSD7A immunohistochemistry was performed as above, except that antigen retrieval was conducted in Tris-EDTA buffer (pH 9.0) using high pressure for 5 minutes. Mouse anti-human THSD7A antibody (amab91233; Sigma) was applied at 1:500 and incubated at 4 °C overnight. A mouse two-step detection kit (PV9002, ZSGB-Bio, Beijing, China) was used, followed by hematoxylin counterstaining, dehydration, mounting, and evaluation at ×200 magnification.

### Statistical analysis

Statistical analyses were performed using SPSS version 20.0 (SPSS Inc., Chicago, IL). Normally distributed variables are presented as mean ± SD and non-normally distributed variables as median (interquartile range, IQR). Categorical variables are presented as n (%). Comparisons between two groups were performed using Student’s t test (normally distributed data) or the Wilcoxon rank-sum test (non-normally distributed data). Comparisons among three or more groups were performed using one-way analysis of variance (ANOVA) or the Kruskal-Wallis test, as appropriate. For categorical variables, the chi-square test or Fisher’s exact test was used. When overall differences were significant, SNK or Dunnett’s tests were used for *post hoc* pairwise comparisons. Receiver operating characteristic (ROC) curves were used to evaluate diagnostic performance. A combined peptide score was generated using logistic regression including the OD values of CysR-11, CTLD7-1, and CTLD7-2. ROC analysis was then performed using this combined score. Because of the modest sample size, this analysis was considered exploratory. All tests were two-sided, and p<0.05 was considered statistically significant.

## Results

### The demographic features and clinical data of patients

**Figure 1:**
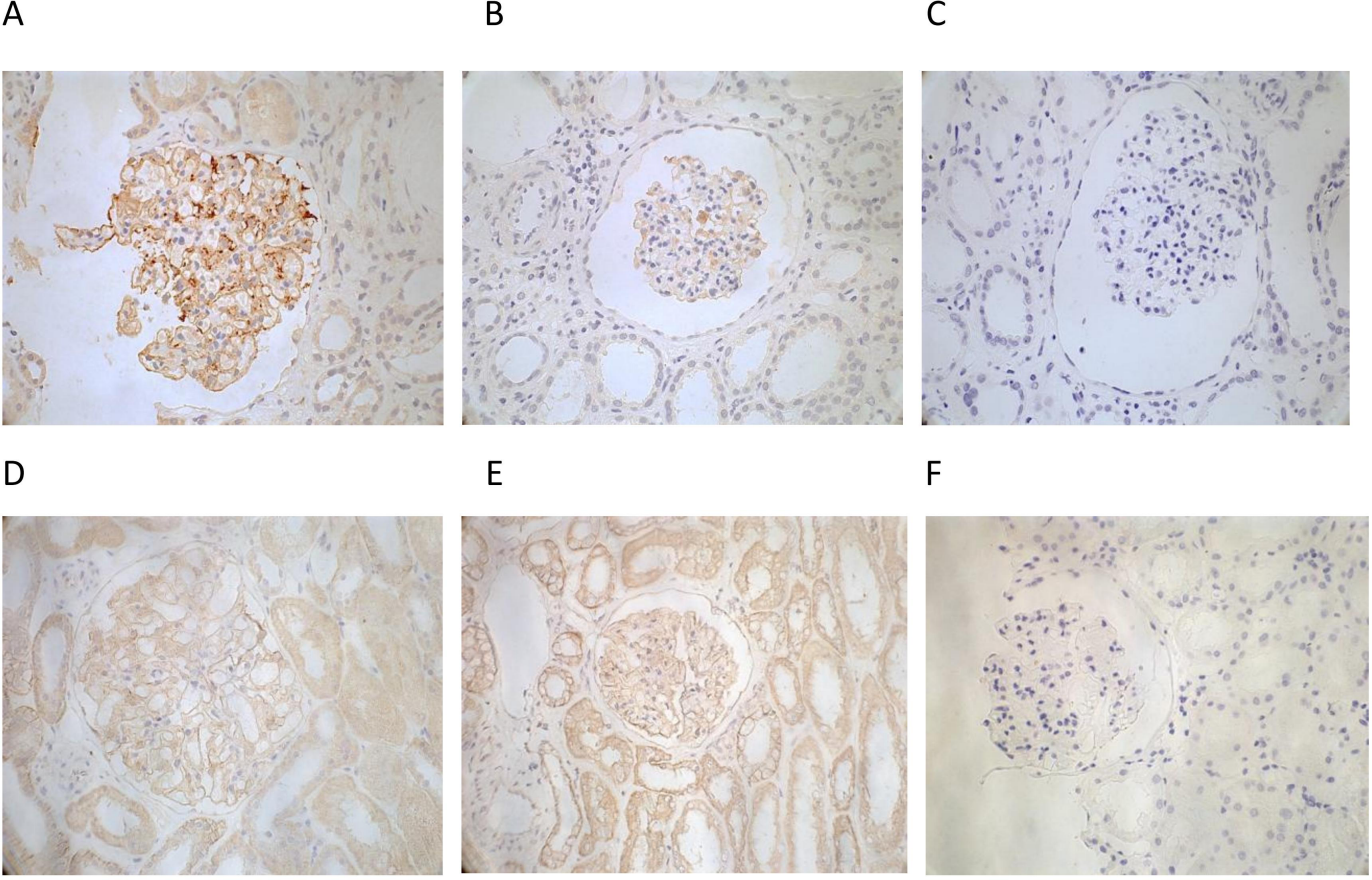
PLA2R and THSD7A staining in kidney tissue. PLA2R staining was positive in biopsy specimens from patients with MN and negative circulating anti-PLA2R antibodies **(A)** but negative in the negative control **(B)** and blank control **(C)**. THSD7A staining was negative in patients with MN and negative circulating anti-PLA2R antibodies **(D)**, the negative control **(E)**, and the blank control **(F)**.

The demographic and clinical characteristics of the 42 patients with PLA2R-associated MN but negative circulating anti-PLA2R antibodies (<14 U/mL) are summarized in **Table 1**. Among them, 33 patients (78.6%) had anti-PLA2R antibody levels below 2 U/mL, 6 patients (14.3%) had levels 2–7 U/mL, and only 3 patients (7.1%) had levels 7–13 U/mL. All patients had granular IgG deposition along the GBM and subepithelial electron-dense deposits. By stage, 45.2% were stage I, 40.5% stage II, and 14.3% stage III; no patient was stage IV. Kidney THSD7A staining was performed in all patients and was negative (**Figure 1**). Other recently described MN target antigens, including NELL1 and EXT1/EXT2, were not systematically evaluated in this cohort because these assays were not routinely available during the study period. All 42 patients were negative for anti-dsDNA antibodies, had no clinical manifestations fulfilling the classification criteria for systemic lupus erythematosus, and had no evidence of hypocomplementemia at diagnosis. .

**Table 1:** Clinical characteristics of patients with PLA2R-associated membranous nephropathy (MN) and negative circulating anti-PLA2R antibodies, comparing those with and without antibodies to PLA2R peptides.

	N=42	With antibodies to PLA2R peptides (n=26)	Without antibodies to PLA2R peptides (n=16)	P value	With antibodies to CysR-11, CTLD7–1 and/or CTLD7-2 (n=15)	Without antibodies to CysR-11, CTLD7–1 and/or CTLD7-2 (n=27)	P value
Age (years) (median, IQR)	50, 33-58	47, 32-61	51, 40-56	0.96	45, 32-55	51, 40-63	0.23
Gender (M/F)	24/18	15/11	9/7	0.93	8/7	16/11	0.71
**Proteinuria (g/24h)**	2.6, 1.5-4.9	2.5, 1.3-4.9	3.3, 2.0-4.7	0.44	**1.5, 1-2.5**	**3.6, 2.4-5.5**	**0.03**
Serum albumin (g/L)	31.3 ± 7.1	31.8 ± 7.0	30.5 ± 7.5	0.57	33.8 ± 6.6	29.9 ± 7.2	0.09
Hematuria, n (%)	19(45.2%)	13(50%)	6(37.5%)	0.43	6(40%)	13(48.1%)	0.61
Serum creatinine (μmol/L)	67.4, 54.4-83.4	67.4, 54.4-83.5	67.1, 52.4-85.4	0.91	62.9, 54.5-87	70.4, 53.9-81.6	0.95
eGFR (ml/min per 1.73m^2^)	104.0, 86.3-119.2	106.0, 77.9-120.3	102.9, 90.0-111.9	0.60	106.6, 71.5-122.8	102.8, 88.9-117.8	0.52
Pathological features							
IgG deposit, n (%)	42(100%)	26(100%)	16(100%)	-	15(100%)	27(100%)	-
IgG1, n (%)	41(97.6%)	25(96.2%)	16(100%)	0.43	15(100%)	26(96.3%)	0.45
IgG2, n (%)	31(73.8%)	21(80.8%)	10(62.5%)	0.20	12(80%)	19(70.4%)	0.50
IgG3, n (%)	20(47.6%)	13(50%)	7(43.8%)	0.70	9(60%)	11(40.7%)	0.23
IgG4, n (%)	40(95.2%)	24(92.3%)	16(100%)	0.26	15(100%)	25(92.6%)	0.28
IgA deposit, n (%)	18(42.9%)	13(50%)	5(31.3%)	0.23	7(46.7%)	11(40.7%)	0.71
IgM deposit, n (%)	13(40.0%)	9(34.6%)	4(25%)	0.51	7(46.7%)	6(22.2%)	0.10
C3 deposit, n (%)	39(92.9%)	24(92.3%)	15(93.8%)	0.86	14(93.3%)	25(92.6%)	0.93
**C1q deposit, n (%)**	14(33.3%)	11(42.3%)	3(18.8%)	0.12	**8(53.3%)**	**6(22.2%)**	**0.04**
PLA2R staining	2, 1-2.5	2, 1.5-2	2, 1-3	0.14	2, 1-2	2, 1-3	0.05
Glomerular lesion							
Stage I, n (%)	19(45.2%)	10(38.5%)	9(56.3%)	0.26	5(33.3%)	14(51.9%)	0.25
Stage II, n (%)	17(40.5%)	12(46.2%)	5(31.3%)	0.34	9(60%)	8(29.6%)	0.06
Stage III, n (%)	6(14.3%)	4(15.4%)	2(12.5%)	0.80	1(6.7%)	5(18.5%)	0.29
Stage IV, n (%)	0(0%)	0(0%)	0(0%)	-	0(0%)	0(0%)	-
**Global sclerosis (%)**	26(61.9%)	**13(50%)**	**13(81.3%)**	**0.04**	**6(40%)**	**20(74.1%)**	**0.03**
Crescent (%)	2(4.8%)	2(7.7%)	0(0%)	0.26	1(6.7%)	1(3.7%)	0.67
Acute tubulointerstitial injury	0(0%)	0(0%)	0(0%)	-	0(0%)	0(0%)	-
Follow-up Treatments	N=29	N=16	N=13		N=9	N=20	
Immunosuppressive agents	15(51.7%)	8(50%)	7(53.9%)	0.84	3(33.3%)	12(60%)	0.18
ACEI/ARBs	28(96.6%)	15(93.8%)	13(100%)	0.36	9(100%)	19(95%)	0.50
Treatment responses							
Clinical remission	26 (89.7%)	15 (93.8%)	11 (84.6%)	0.42	9 (100%)	17 (85%)	0.22
Complete remission	17(58.6%)	10(62.5%)	7(53.8%)	0.64	6(66.7%)	11(55%)	0.56
Partial remission	9(31.0%)	5(31.3%)	4(30.8%)	0.98	3(33.3%)	6(30.0%)	0.86
No remission	3(10.3%)	1(6.3%)	2(15.4%)	0.42	0(0%)	3(15.0%)	0.22
Relapse	3/26(11.5%)	1/15(6.7%)	2/11(18.2%)	0.42	1/9(11.1%)	2/17(11.8%)	0.93
Kidney outcomes							
ESKD	0(0%)	0(0%)	0(0%)	-	0(0%)	0(0%)	-
eGFR reduction >50%	0(0%)	0(0%)	0(0%)	-	0(0%)	0(0%)	-
Follow-up (months)	36.0, 18.5-48.0	34.0, 20.5-47.8	36.0, 12.5-48.0	0.84	44.0, 28.5-51.0	33.5, 12.3-47.3	0.10

Continuous and normally distributed variables were presented as mean ± SD; continuous and non-normally distributed variables were presented as median, IQR; categorical variables were presented as n (%); the intensity of immunohistochemistry +, ++, +++ were presented as numbers. Bold values represent significant differences (P < 0.05).

### Antibodies towards PLA2R peptides in seronegative PLA2R-associated MN

We synthesized 123 overlapping linear peptides spanning the extracellular region of PLA2R and assessed circulating IgG reactivity to these peptides by ELISA in patients with PLA2R-associated MN (anti-PLA2R-negative and anti-PLA2R-positive), secondary MN (HBV- and tumor-associated), disease controls (MCD, FSGS, IgA nephropathy), and healthy controls.

Among the 42 seronegative PLA2R-associated MN patients, plasma from 26 (61.9%) reacted with at least one PLA2R peptide. Of these, 24 (57.1%) recognized 1–8 peptides, and two (4.8%) recognized more than 10 peptides. Sixteen patients (38.1%) showed no reactivity to any peptide.

**Figure 2:**
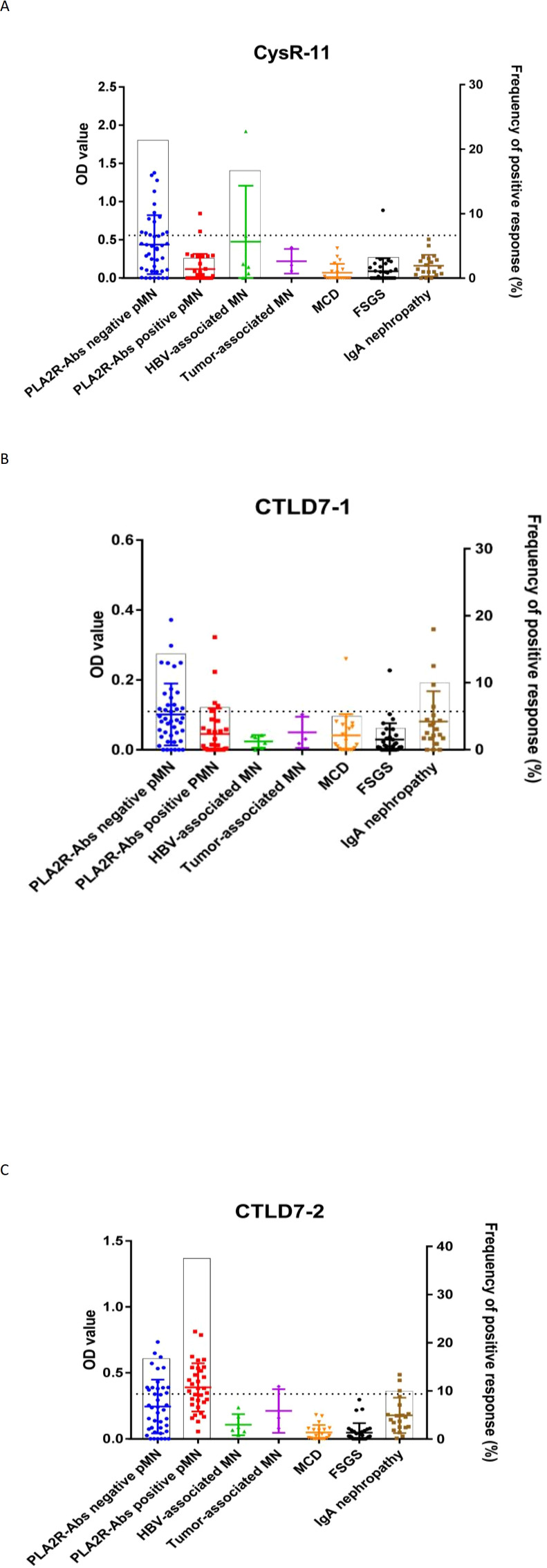
Reactivity to PLA2R peptides CysR-11 **(A)**, CTLD7-1 **(B)**, and CTLD7-2 **(C)** across disease groups. Dots indicate the optical density (OD) value for each participant. The dotted line indicates the cut-off value (mean + 2 SD) derived from 40 healthy donors. Bars show the frequency of positive reactions in each group.

Across the 123 peptides, 68 were recognized by plasma from anti-PLA2R-negative MN patients. Three peptides showed the highest recognition frequencies: CysR-11 (21.4%), CTLD7-1 (14.3%), and CTLD7-2 (16.7%) (**Table 2**; **Figure 2**). Recognition frequencies for other peptides ranged from 2.4% to 9.5%, and 55 peptides were not recognized.

**Table 2:** Most frequently recognized PLA2R peptides in PLA2R-associated MN patients.

Peptide	With negative anti-PLA2R antibodies		With positive anti-PLA2R antibodies		Cut-off values
	Recognition rate, n (%)	Antibody levels, Mean ± SD	Recognition rate, n (%)	Antibody levels, Mean ± SD	
CysR-5	2 (4.8)	0.63 ± 0.01	4 (12.5)	1.16 ± 0.59	0.51
CysR-11	9 (21.4)	1.03 ± 0.26	1 (3.1)	0.85	0.46
CTLD1-7	1 (2.4)	0.42	5 (15.6)	0.38 ± 0.11	0.18
CTLD4-7	0 (0)	0	7 (21.9)	1.28 ± 0.43	0.58
CTLD5-6	0 (0)	0	6 (18.8)	0.58 ± 0.29	0.24
CTLD5-9	4 (9.5)	0.42 ± 0.05	4 (12.5)	0.43 ± 0.33	0.28
CTLD6-12	0 (0)	0	5 (15.6)	0.66 ± 0.18	0.43
CTLD7-1	6 (14.3)	0.28 ± 0.05	2 (6.3)	0.27 ± 0.07	0.11
CTLD7-2	7 (16.7)	0.58 ± 0.1	12 (37.5)	0.58 ± 0.12	0.34

The main table includes peptides with a recognition frequency ≥10% in either the anti-PLA2R-negative or anti-PLA2R-positive MN group, as well as the candidate peptides CysR-11, CTLD7-1, and CTLD7-2. The full 123-peptide dataset is provided in **Supplementary Table 2**.

### Antibodies towards PLA2R peptides in anti-PLA2R positive MN

Among the 32 anti-PLA2R-positive MN patients, plasma from 28 (87.5%) reacted with at least one PLA2R peptide. Of these, 26 (81.3%) recognized 1–9 peptides and two (6.3%) recognized more than 10 peptides; four patients (12.5%) showed no reactivity.

In anti-PLA2R-positive patients, 53 PLA2R peptides were recognized. Seven peptides showed relatively high recognition frequencies: CysR-5 (12.5%), CTLD1-7 (15.6%), CTLD4-7 (21.9%), CTLD5-6 (18.8%), CTLD5-9 (12.5%), CTLD6-2 (15.6%), and CTLD7-2 (37.5%) (**Table 2**). Recognition frequencies for other peptides ranged from 3.1% to 9.4%, and 70 peptides were not recognized.

### Antibodies towards PLA2R peptides in secondary MN

We also included six patients with HBV-associated MN and three with tumor-associated MN. All were negative for circulating anti-PLA2R antibodies but showed PLA2R expression in kidney tissue. THSD7A staining was negative in all cases.

Five of six patients (83.3%) with HBV-associated MN reacted with PLA2R peptides. One patient recognized three peptides, three patients recognized two peptides, and one patient recognized one peptide; one patient showed no reactivity. CTLD4–10 and CTLD7-7–1 were recognized in 33.3%, and CysR-11, CysR-12, CTLD1-4, CTLD5-9, CTLD7-7-2, and CTLD8-4–2 were each recognized in 16.7%.

Two of three patients (66.7%) with tumor-associated MN reacted with PLA2R peptides. One patient recognized 33 peptides and one recognized nine peptides; one patient showed no reactivity. Thirty-four peptides were recognized in 33.3% of patients and eight peptides were recognized in 66.7%.

### Comparisons of antibodies towards PLA2R peptides among groups

CysR-11, CTLD7-1, and CTLD7–2 showed the highest recognition frequencies in anti-PLA2R-negative MN (**Figure 2**). The recognition frequency of CysR-11 was significantly higher in anti-PLA2R-negative than in anti-PLA2R-positive MN (21.4% vs. 3.1%, p=0.023) and was also higher than in MCD (0%, p=0.025), FSGS (3.2%, p=0.025), and IgA nephropathy (0%, p=0.025). Differences versus tumor-associated MN (0%, p=0.37) and HBV-associated MN (16.7%, p=0.788) were not significant.

The recognition frequency of CTLD7–1 was 14.3% in seronegative PLA2R-associated MN and was higher than in anti-PLA2R-positive MN (6.3%, p=0.27), MCD (0%, p=0.28), FSGS (3.2%, p=0.113), tumor-associated MN (0%, p=0.48), and HBV-associated MN (0%, p=0.322), but similar to IgA nephropathy (10%, p=0.638).

The recognition frequency of CTLD7–2 was 16.7% in seronegative PLA2R-associated MN. This was lower than in anti-PLA2R-positive MN (37.5%, p=0.04) but higher than in MCD (0%, p=0.053) and FSGS (0%, p=0.02), and similar to IgA nephropathy (10%, p=0.486), tumor-associated MN (0%, p=0.442), and HBV-associated MN (0%, p=0.279).

### Exploratory performance of PLA2R peptide reactivity in seronegative PLA2R-associated MN

**Figure 3:**
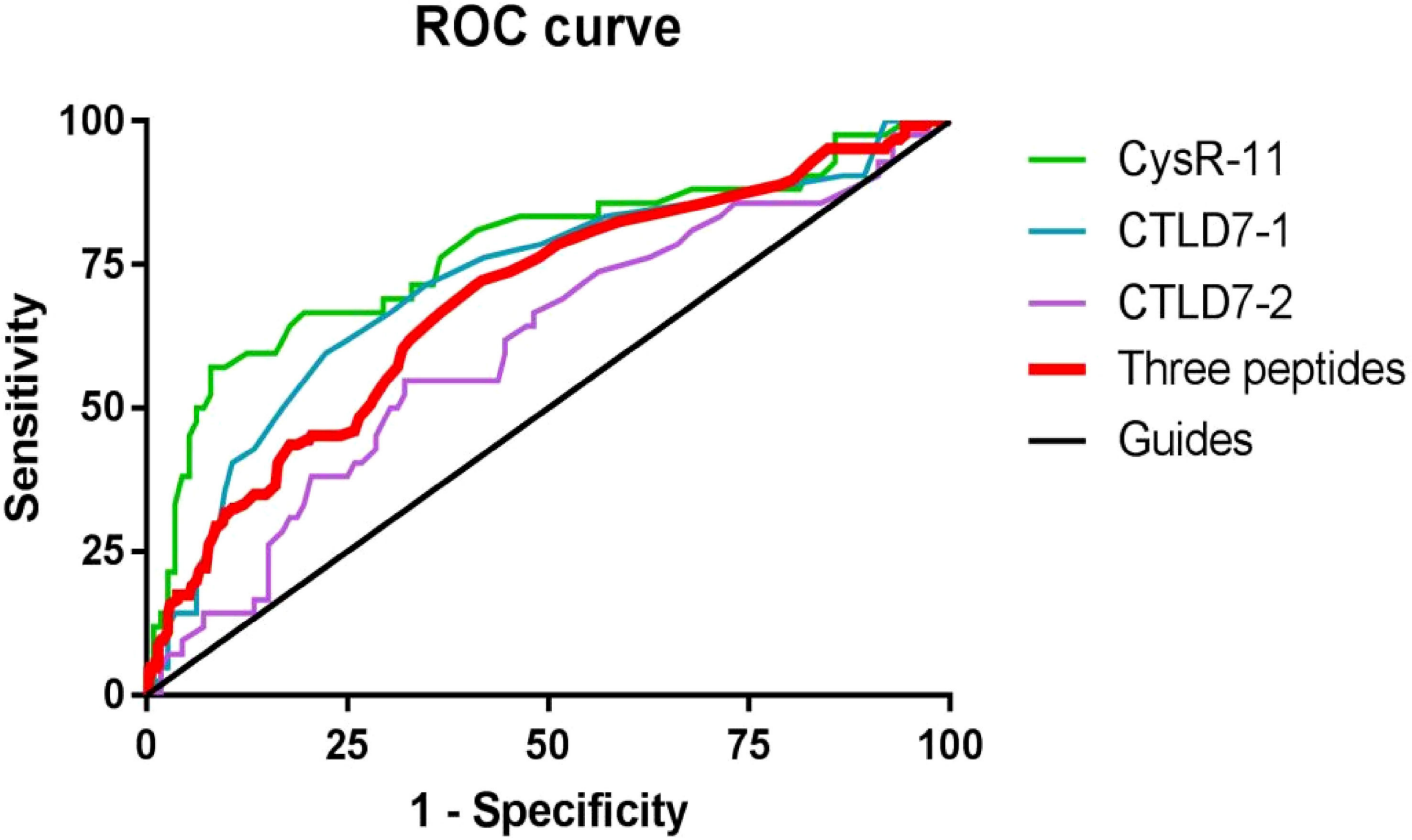
ROC curves for peptide ELISA in diagnosing PLA2R-associated MN with negative circulating anti-PLA2R antibodies. P values for CysR-11, CTLD7-1, and CTLD7–2 were all <0.05. Combined (any positive) detection of the three peptides yielded 43.7% sensitivity and 81.3% specificity.

The area under the ROC curve (AUC) for CysR-11 was 0.770 (95% CI 0.677-0.862; p<0.001), with 38.1% sensitivity and 94.7% specificity. The AUC for CTLD7–1 was 0.716 (95% CI 0.621-0.811; p<0.001), with 2.4% sensitivity and 100% specificity. The AUC for CTLD7–2 was 0.605 (95% CI 0.504-0.706; p=0.044), with 50.0% sensitivity and 68.8% specificity. Combining CysR-11, CTLD7-1, and CTLD7–2 if any of them was positive yielded 43.7% sensitivity and 81.3% specificity (AUC = 0.685, 95% CI 0.630-0.740; p<0.001), indicating moderate diagnostic performance. These findings suggest that peptide reactivity may provide additional serological information in selected patients with seronegative PLA2R-associated MN, but the assay is not suitable as a stand-alone diagnostic test (**Figure 3**).

### Assessment of competitive inhibition between PLA2R peptides and full-length PLA2R

**Figure 4:**
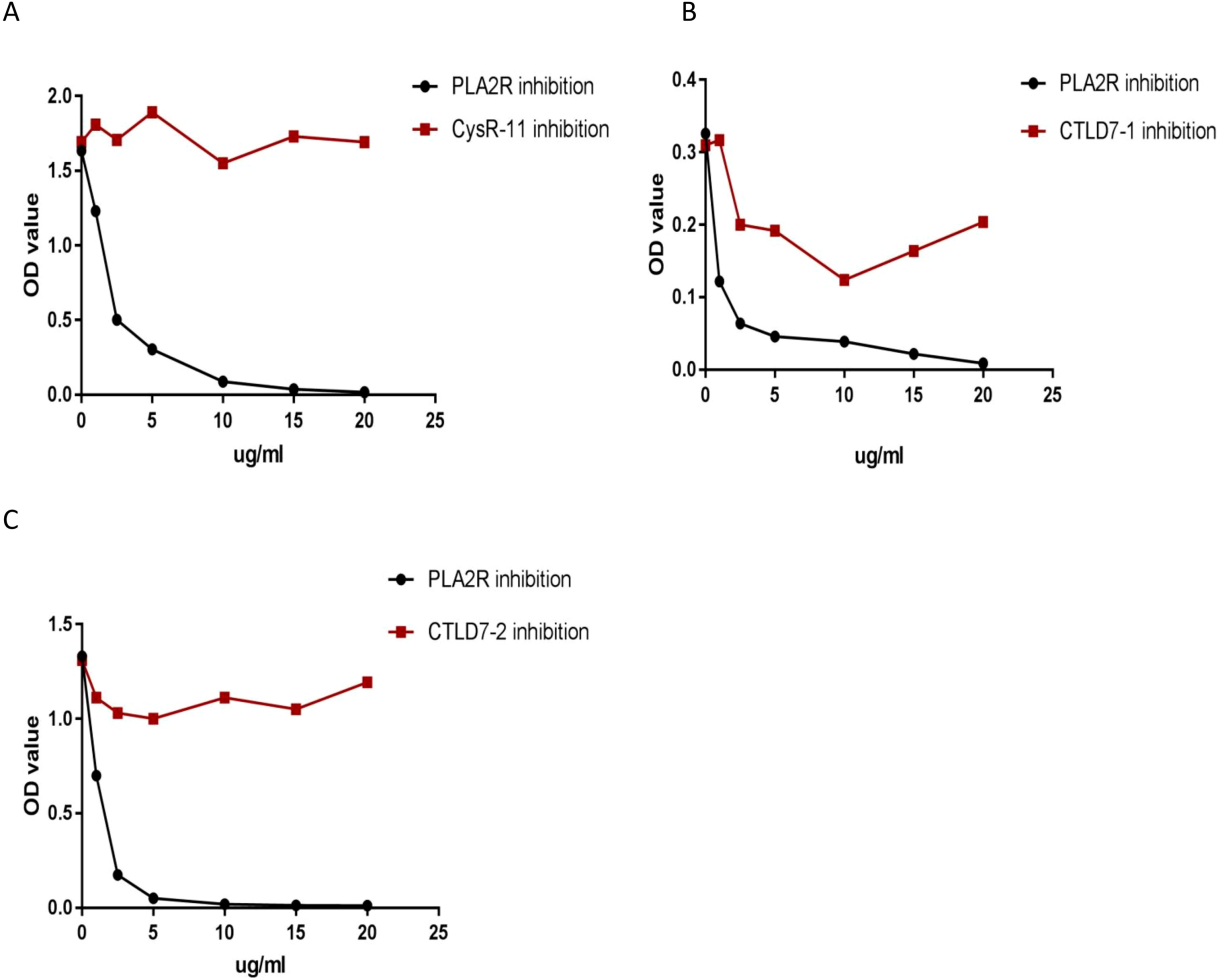
Inhibition ELISA assessing competition between PLA2R peptides and full-length PLA2R. Binding of circulating anti-PLA2R antibodies to coated full-length PLA2R was inhibited by preincubation with soluble PLA2R protein, but was not detectably inhibited by CysR-11 **(A)**, CTLD7-1 **(B)**, or CTLD7–2 **(C)** under the experimental conditions used.

CysR-11, CTLD7-1, and CTLD7–2 were used as inhibitors in ELISA to assess cross-reactivity with anti-PLA2R antibodies. Binding of anti-PLA2R antibodies to coated full-length PLA2R was inhibited by preincubation with soluble PLA2R protein, but not detectably inhibited by CysR-11, CTLD7-1, or CTLD7–2 under the experimental conditions used. These findings suggest that the three short linear peptides did not compete with full-length PLA2R for binding to circulating anti-PLA2R antibodies in this assay. However, this result does not exclude partial overlap, low-affinity interactions, or differences in epitope accessibility (**Figure 4**).

### Clinical characteristics of the patients recognizing PLA2R peptides

The 42 seronegative PLA2R-associated MN patients were stratified by the presence of antibodies to any PLA2R peptide: 26 had peptide-reactive antibodies and 16 did not (**Table 1**). Patients with peptide reactivity were 47 (32–61) years old, with a male/female ratio of 15/11. They presented with proteinuria of 2.5 (1.3-4.9) g/24 h, serum albumin of 31.8 ± 7.0 g/L, and eGFR of 106.0 (77.9-120.3) mL/min/1.73m2. Kidney PLA2R staining intensity was 2+ (1.5-2), and IgG deposits were predominantly IgG4 and IgG1. Fifteen patients had antibodies to CysR-11, CTLD7-1, and/or CTLD7-2 (**Table 1**). Compared with those without these antibodies, they had lower proteinuria (1.5 vs. 3.6 g/24 h, p=0.03), a higher frequency of C1q deposition (53.3% vs. 22.2%, p=0.04), and a lower frequency of global sclerosis (40.0% vs. 74.1%, p=0.03). Given the limited sample size and multiple comparisons, these findings should be interpreted cautiously and require validation in independent cohorts.

Twenty-nine seronegative PLA2R-associated MN were followed for 36.0 (18.5-48.0) months. Almost all (96.6%) received renin-angiotensin-aldosterone system inhibitors and 51.7% received immunosuppressive therapy. Clinical remission was achieved in 26 patients (89.7%), including complete remission in 17 (58.6%) and partial remission in nine (31.0%). Treatment responses, relapse rates, ESKD, and >50% eGFR reduction did not differ significantly between patients with and without peptide-reactive antibodies (**Table 1**). Therefore, baseline peptide reactivity was not associated with a clear prognostic signal in this cohort.

## Discussion

In this study, we screened patients with biopsy-proven PLA2R-associated MN who were negative for circulating anti-PLA2R antibodies and found that 61.9% had detectable IgG reactivity to at least one linear PLA2R peptide. This suggests that PLA2R-directed autoimmunity is present in a substantial fraction of seronegative PLA2R-associated MN patients, even when antibodies to the native conformational antigen are not detected. Reported proportions of seronegative PLA2R-associated MN range from 18% to 41% across cohorts (24–26). For this subgroup, there is currently no established circulating biomarker to reflect immunologic activity analogous to anti-PLA2R antibodies in seropositive disease (27). Our results identify three peptides, CysR-11, CTLD7-1, and CTLD7-2, that showed relatively higher recognition in seronegative patients. However, the combined sensitivity and specificity of these peptides were only moderate. Therefore, these findings should be interpreted as exploratory and hypothesis-generating. Peptide-reactive antibodies may provide complementary serological information in a subset of patients with anti-PLA2R-negative PLA2R-associated MN, but they are not intended to replace kidney biopsy or kidney PLA2R staining for initial diagnosis. Their potential value for longitudinal monitoring remains to be determined in future studies with serial sampling.

We also detected antibodies to PLA2R peptides in patients with circulating anti-PLA2R antibodies, with an even higher proportion showing peptide reactivity (87.5%), which is consistent with other autoimmune kidney diseases characteristic of autoantibodies (16, 28, 29). However, the peptide recognition spectrum differed between seronegative and seropositive patients. CysR-11 and CTLD7–1 were enriched in seronegative disease, whereas several other peptides (e.g., CysR-5, CTLD1-7, CTLD4-7, CTLD5-6, CTLD5-9, CTLD6-2, and CTLD7-2) were more frequently recognized in seropositive disease. This divergence suggests that immunologic trajectories may differ between these subgroups. Although seronegative PLA2R-associated MN is often considered an early stage reflecting the ‘kidney as a sink’ phenomenon (30, 31), some patients show severe clinical disease without detectable circulating anti-PLA2R antibodies (32), and correlations between anti-PLA2R titers and proteinuria are imperfect (33, 34). Peptide-reactive antibodies and/or autoreactive T cells may therefore contribute to kidney injury in seronegative PLA2R-associated MN, analogous to mechanisms described in anti-GBM disease and ANCA-associated vasculitis (35, 36). Further mechanistic studies are warranted.

**Figure 5:**
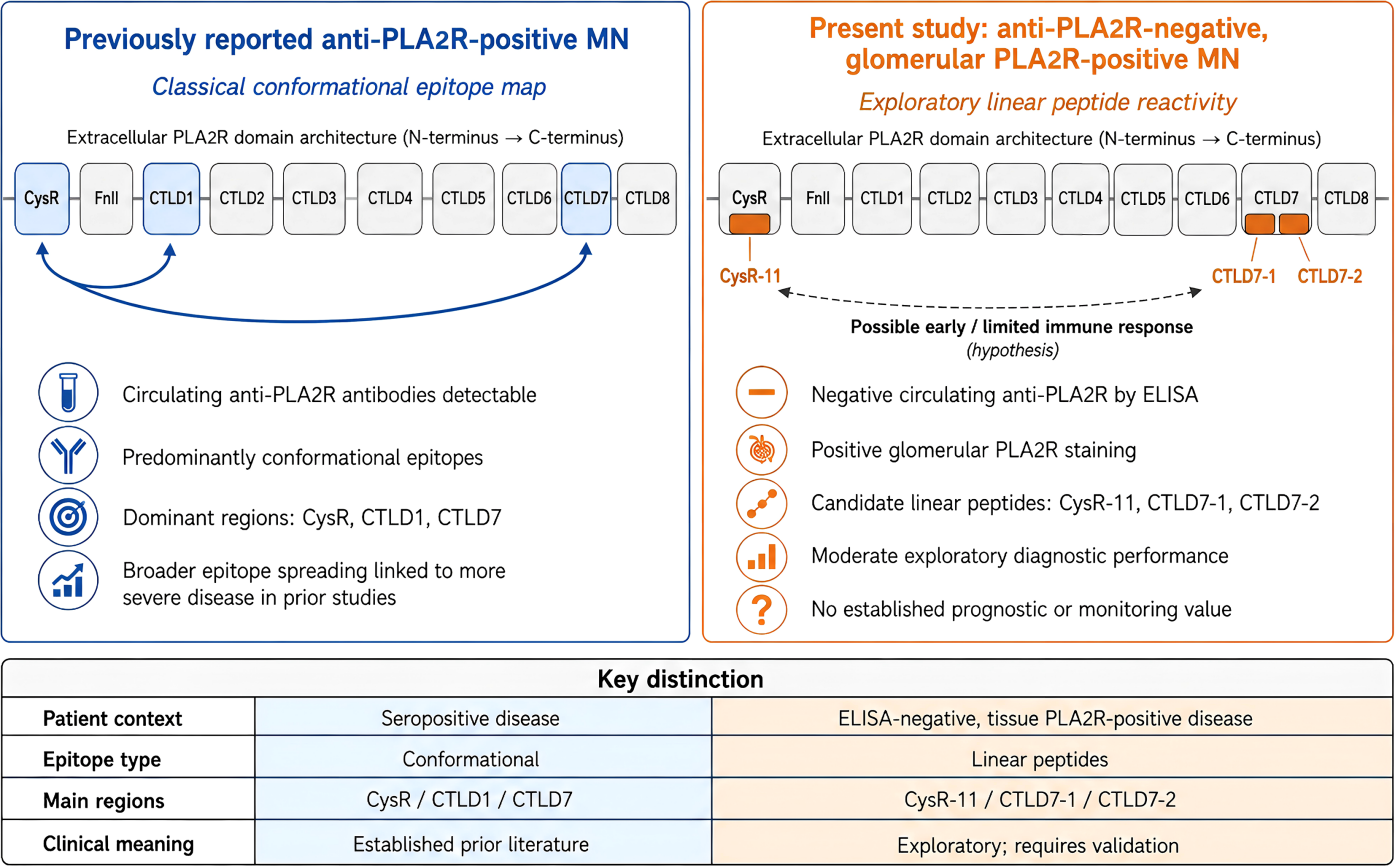
Schematic comparison of PLA2R epitope patterns in MN. The left panel summarizes previously reported conformational epitopes in anti-PLA2R-positive MN, mainly involving CysR, CTLD1, and CTLD7. The right panel shows the exploratory linear peptide reactivity identified in the present anti-PLA2R-negative, glomerular PLA2R-positive MN cohort, highlighting CysR-11, CTLD7-1, and CTLD7-2. This schematic emphasizes that the peptide-reactive pattern observed here is distinct from the classical conformational epitope-spreading pattern described in seropositive disease.

Previous studies have focused largely on conformational epitopes in CysR and on epitope spreading to CTLD1 and CTLD7 (9–12) (**Figure 5**). In particular, two CysR-derived peptides showed strong inhibitory activity, and a 31-mer spanning these sequences inhibited binding of autoantibodies to PLA2R (12). We tested the corresponding linear peptides (CysR-1 and CysR-3) but did not detect direct antibody binding in either seronegative or seropositive patients, supporting the notion that dominant anti-PLA2R epitopes depend on three-dimensional structure. In contrast, the candidate linear peptides identified in our study did not detectably inhibit the binding of circulating anti-PLA2R antibodies to full-length PLA2R in inhibition ELISA. This suggests that, under the assay conditions used, these short peptides did not substantially compete with the intact antigen for anti-PLA2R antibody binding. However, this finding should not be interpreted as definitive evidence that peptide-reactive antibodies and antibodies to intact PLA2R are entirely unrelated. Partial epitope overlap, low-affinity binding, conformational dependence, or altered epitope accessibility cannot be excluded by this assay.

The biological roles of these peptide-reactive antibodies remain to be elucidated. One possible explanation is that anti-peptide antibodies may precede detectable antibodies against intact PLA2R. In this context, reactivity to linear PLA2R peptides may represent an early stage of intramolecular epitope spreading, before the development of broader, higher-affinity, or conformational anti-PLA2R antibody responses. Such an early immune phase could be associated with milder clinical manifestations and fewer chronic histological lesions, as observed in our peptide-reactive subgroup. However, because our study was cross-sectional and lacked serial samples obtained before and after anti-PLA2R seroconversion, we cannot prove the temporal sequence between anti-peptide antibodies and antibodies to intact PLA2R. This hypothesis requires validation in prospective longitudinal cohorts.

This study has limitations. First, screening reactivity to 123 peptides required large sample volumes, limiting the number of patients with HBV-associated or tumor-associated MN available for analysis. Second, although most patients were followed for approximately three years, the high remission rate and absence of kidney function decline limited our ability to assess associations between peptide-reactive antibodies and long-term outcomes. Larger cohorts with longer follow-up and serial sampling will be needed to evaluate prognostic value. Third, although we evaluated the baseline presence of peptide-reactive antibodies, serial samples were not available for most patients. Therefore, we could not determine whether peptide antibody levels change with disease activity, treatment response, or relapse. The potential role of these antibodies in longitudinal monitoring remains speculative and requires prospective validation.

In conclusion, antibodies to linear PLA2R peptides are detectable in a subset of PLA2R-associated MN patients who are seronegative for circulating anti-PLA2R antibodies. CysR-11, CTLD7-1, and CTLD7–2 showed relatively higher recognition in this subgroup, but their combined diagnostic performance was moderate, and their clinical utility remained unproven. These peptides should therefore be considered exploratory candidate serological markers that may provide complementary information in selected patients. Larger prospective studies with serial sampling are needed to determine whether peptide-reactive antibodies track disease activity or treatment response.

## Data Availability

The original contributions presented in the study are included in the article/**Supplementary Material**. Further inquiries can be directed to the corresponding author.
