# Supplementary material for: Identification of antibodies against phospholipase A2 receptor peptides in PLA2R-associated membranous nephropathy with negative circulating anti-PLA2R antibodies
Source: Front Immunol. 2026 May 28;17:1816719. doi: 10.3389/fimmu.2026.1816719 (PMC13253692; doi:10.3389/fimmu.2026.1816719)
Supplement: Supplementary file 1 [file DataSheet1.pdf]

Supplementary Table. PLA2R linear peptides

Peptide	Position from N-terminal	Sequence from N-terminal
CysR-1	38-52	KGIFVIQSESLKKCI
CysR-2	45-59	SESLKKCIQAGKSVL
CysR-3	52-66	IQAGKSVLTLENCKQ
CysR-4	59-73	LTLENCKQANKHMLW
CysR-5	64-80	CKQANKHMLWKWVSNHG
CysR-6	71-87	MLWKWVSNHGLFNIGGS
CysR-7	80-94	GLFNIGGSGCLGLNF
CysR-8	85-101	GGSGCLGLNFSAPEQPL
CysR-9	94-108	FSAPEQPLSLYECDS
CysR-10	101-120	LSLYECDSTLVSLRWRCNRK
CysR-11	108-122	STLVSLRWRCNRKMI
CysR-12	113-129	LRWRCNRKMITGPLQYS
CysR-13	122-136	ITGPLQYSVQVAHDN
CysR-14	129-143	SVQVAHDNTVVASRK
CysR-15	136-150	NTVVASRKYIHKWIS
CysR-16	141-157	SRKYIHKWISYGSGGGD
CysR-17	150-161	SYGSGGGDICEY
FnII-1	173-192	THGMPCMFPFQYNHQWHHEC
FnII-2	183-202	QYNHQWHHECTREGREDDLL
FnII-3	193-212	TREGREDDLLWCATTSRYER
FnII-4	203-221	WCATTSRYERDEKWGFCPD
CTLD1-1-1	138-247	NSHICYQFNL
CTLD1-1-2	246-257	NLLSSLSWSEAH
CTLD1-2	248-267	LSSLSWSEAHSSCQMQGGTL
CTLD1-3	258-277	SSCQMQGGTLLSITDETEEN
CTLD1-4	268-287	LSITDETEENFIREHMSSKT
CTLD1-5	278-297	FIREHMSSKTVEVWMGLNQL
CTLD1-6	288-307	VEVWMGLNQLDEHAGWQWSD
CTLD1-7	298-317	DEHAGWQWSDGTPLNYLNWS
CTLD1-8	308-327	GTPLNYLNWSPEVNFEPFVE
CTLD1-9	318-337	PEVNFEPFVEDHCGTFSSFM
CTLD1-10	328-347	DHCGTFSSFMPSAWRSRDCE
CTLD1-11	338-355	PSAWRSRDCESTLPYICK
CTLD2-1	385-404	YNRNCYKLQKEEKTWHEALR
CTLD2-2	395-414	EEKTWHEALRSCQADNSALI
CTLD2-3	405-424	SCQADNSALIDITSLAEVEF
CTLD2-4-1	415-424	DITSLAEVEF
CTLD2-4-2	425-434	LVTLLGDENA
CTLD2-5	425-444	LVTLLGDENASETWIGLSSN
CTLD2-6	435-454	SETWIGLSSNKIPVSFEWSN
CTLD2-7	445-464	KIPVSFEWSNDSSVIFTNWH
CTLD2-8	455-474	DSSVIFTNWHTLEPHIFPNR

CTLD2-9	465-484	TLEPHIFPNRSQLCVSAEQS
CTLD2-10	475-494	SQLCVSAEQSEGHWKVKNCE
CTLD2-11	485-502	EGHWKVKNCEERLFYICK
CTLD3-1	520-541	ERHGGFCYKIDTVLRSFDQASS
CTLD3-2	532-551	VLRSFDQASSGYYCPPALVT
CTLD3-3	542-561	GYYCPPALVTITNRFEQAFI
CTLD3-4	550-571	VTITNRFEQAFITSLISSVVKM
CTLD3-5	562-581	TSLISSVVKMKDSYFWIALQ
CTLD3-6	570-591	KMKDSYFWIALQDQNDTGEYTW
CTLD3-7	582-601	DQNDTGEYTWKPVGQKPEPV
CTLD3-8	592-611	KPVGQKPEPVQYTHWNTHQP
CTLD3-9	602-621	QYTHWNTHQPRYSGGCVAMR
CTLD3-10	612-631	RYSGGCVAMRGRHPLGRWEV
CTLD3-11	622-641	GRHPLGRWEVKHCRHFKAMS
CTLD3-12	632-643	KHCRHFKAMSLC
CTLD4-1	673-692	GLASCFKVFHSEKVLMKRTW
CTLD4-2	683-702	SEKVLMKRTWREAEAFCEEF
CTLD4-3	693-712	REAEAFCEEFGAHLASFAHI
CTLD4-4	703-722	GAHLASFAHIEEENFVNELL
CTLD4-5	713-732	EEENFVNELLHSKFNWTEER
CTLD4-6	723-742	HSKFNWTEERQFWIGFNKRN
CTLD4-7	731-752	ERQFWIGFNKRNPLNAGSWEWS
CTLD4-8	743-762	PLNAGSWEWSDRTPVVSSFL
CTLD4-9	753-772	DRTPVVSSFLDNTYFGEDAR
CTLD4-10	763-782	DNTYFGEDARNCAVYKANKT
CTLD4-11	773-792	NCAVYKANKTLLPLHCGSKR
CTLD4-12	783-797	LLPLHCGSKREWICK
CTLD5-1	819-838	YQDAEYLFHTFASEWLNFEF
CTLD5-2-1	829-838	FASEWLNFEF
CTLD5-2-2	837-848	EFVCSWLHSDLL
CTLD5-3	837-858	EFVCSWLHSDLLTIHSAHEQEF
CTLD5-4	849-868	TIHSAHEQEFIHSKIKALSK
CTLD5-5	859-878	IHSKIKALSKYGASWWIGLQ
CTLD5-6	869-888	YGASWWIGLQEERANDEFRW
CTLD5-7	879-898	EERANDEFRWRDGTPVIYQN
CTLD5-8	889-908	RDGTPVIYQNWDTGRERTVN
CTLD5-9	899-918	WDTGRERTVNNQSQRCGFIS
CTLD5-10	909-928	NQSQRCGFISSITGLWGSEE
CTLD5-11	919-938	SITGLWGSEECSVSMPSICK
CTLD6-1	963-984	LYFNYKCLLLNIPKDPSSWKNW
CTLD6-2	975-994	PKDPSSWKNWTHAQHFCAEE
CTLD6-3	985-1004	THAQHFCAEEGGTLVAIESE
CTLD6-4-1	993-1004	EEGGTLVAIESE
CTLD6-4-2	1005-1014	VEQAFITMNL

CTLD6-5	1005-1024	VEQAFITMNLFGQTTSVWIG
CTLD6-6-1	1015-1024	FGQTTSVWIG
CTLD6-6-2	1025-1034	LQNDDYETWL
CTLD6-7	1025-1044	LQNDDYETWLNGKPVVYSNW
CTLD6-8	1033-1054	WLNGKPVVYSNWSPFDIINIPS
CTLD6-9	1045-1064	SPFDIINIPSHNTTEVQKHI
CTLD6-10	1055-1074	HNTTEVQKHIPLCALLSSNP
CTLD6-11	1065-1084	PLCALLSSNPNFHFTGKWYF
CTLD6-12	1075-1094	NFHFTGKWYFEDCGKEGYGF
CTLD6-13	1085-1096	EDCGKEGYGFVC
CTLD7-1	1121-1140	YGNRTYKIINANMTWYAAIK
CTLD7-2	1129-1150	INANMTWYAAIKTCLMHKAQLV
CTLD7-3	1139-1160	IKTCLMHKAQLVSITDQYHQSF
CTLD7-4	1151-1170	SITDQYHQSFLTVVLNRLGY
CTLD7-5-1	1159-1170	SFLTVVLNRLGY
CTLD7-5-2	1169-1180	GYAHWIGLFTTD
CTLD7-6	1171-1190	AHWIGLFTTDNGLNFDWSDG
CTLD7-7-1	1181-1190	NGLNFDWSDG
CTLD7-7-2	1189-1200	DGTKSSFTFWKD
CTLD7-8	1191-1210	TKSSFTFWKDEESSLLGDCV
CTLD7-9	1201-1220	EESSLLGDCVFADSNGRWHS
CTLD7-10	1211-1230	FADSNGRWHSTACESFLQGA
CTLD7-11	1221-1232	TACESFLQGAIC
CTLD8-1-1	1255-1266	IKFKSNCYSFST
CTLD8-1-2	1267-1276	VLDSMSFEAA
CTLD8-2	1267-1286	VLDSMSFEAAHEFCKKEGSN
CTLD8-3	1277-1296	HEFCKKEGSNLLTIKDEAEN
CTLD8-4-1	1287-1296	LLTIKDEAEN
CTLD8-4-2	1295-1306	ENAFLLEELFAF
CTLD8-5-2	1307-1316	GSSVQMVWLN
CTLD8-6-2	1317-1326	AQFDGNNETI
CTLD8-7	1317-1336	AQFDGNNETIKWFDGTPTDQ
CTLD8-8	1327-1346	KWFDGTPTDQSNWGIRKPDT
CTLD8-9	1337-1356	SNWGIRKPDTDYFKPHHCVA
CTLD8-10	1345-1366	DTDYFKPHHCVALRIPEGLWQL
CTLD8-11	1357-1376	LRIPEGLWQLSPCQEKKGFI
CTLD8-12	1367-1378	SPCQEKKGFICK
