# Supplementary material for: Identification of antibodies against phospholipase A2 receptor peptides in PLA2R-associated membranous nephropathy with negative circulating anti-PLA2R antibodies
Source: Front Immunol. 2026 May 28;17:1816719. doi: 10.3389/fimmu.2026.1816719 (PMC13253692; doi:10.3389/fimmu.2026.1816719)
Supplement: Supplementary file 2 [file DataSheet2.pdf]

Supplementary Table 2.PLA2R peptides recognized by the MN patients.

Peptide	Recognition rate in MN with negative anti-PLA2R antibodies, n (%)	Antibody levels, Mean ± SD	Recognition rate in MN with positive anti-PLA2R antibodies, n (%)	Antibody levels, Mean ± SD	Cut-off values
CysR-1	2 (4.8)	0.73±0.10	1 (3.1)	0.64	0.44
CysR-2	3 (7.1)	0.63±0.07	3 (9.4)	1.05±0.54	0.50
CysR-3	1 (2.4)	0.76	0 (0)	0	0.30
CysR-4	1 (2.4)	0.76	0 (0)	0	0.61
CysR-5	2 (4.8)	0.63±0.01	4 (12.5)	1.16±0.59	0.51
CysR-6	1 (2.4)	0.46	0 (0)	0	0.27
CysR-7	0 (0)	0	2 (6.3)	0.48±0.06	0.26
CysR-8	0 (0)	0	1 (3.1)	0.33	0.23
CysR-9	1 (2.4)	0.46	1 (3.1)	0.62	0.05
CysR-10	1 (2.4)	1.23	1 (3.1)	1.03	0.80
**CysR-11**	**9 (21.4)**	**1.03±0.26**	**1 (3.1)**	**0.85**	**0.46**
CysR-12	1 (2.4)	0.92	0 (0)	0	0.75
CysR-13	1 (2.4)	0.30	2 (6.3)	0.33±0.01	0.19
CysR-14	0 (0)	0	0 (0)	0	0.34
CysR-15	0 (0)	0	0 (0)	0	0.76
CysR-16	1 (2.4)	0.81	1 (3.1)	0.92	0.56
CysR-17	0 (0)	0	0 (0)	0	0.44
FnII-1	1 (2.4)	0.58	0 (0)	0	0.47
FnII-2	1 (2.4)	0.54	3 (9.4)	0.19±0.02	0.06
FnII-3	0 (0)	0	0 (0)	0	0.26
FnII-4	1 (2.4)	0.46	0 (0)	0	0.25
CTLD1-1-1	2 (4.8)	0.47±0.11	1 (3.1)	0.30	0.16
CTLD1-1-2	1 (2.4)	0.40	0 (0)	0	0.11
CTLD1-2	1 (2.4)	0.52	0 (0)	0	0.42
CTLD1-3	0 (0)	0	0 (0)	0	0.00
CTLD1-4	3 (7.1)	0.36±0.07	1 (3.1)	0.78	0.12
CTLD1-5	0 (0)	0	0 (0)	0	0.19
CTLD1-6	0 (0)	0	1 (3.1)	0.70	0.28
CTLD1-7	1 (2.4)	0.42	5 (15.6)	0.38±0.11	0.18
CTLD1-8	2 (4.8)	0.35±0.22	1 (3.1)	0.29	0.09
CTLD1-9	1 (2.4)	0.40	1 (3.1)	0.24	0.11
CTLD1-10	2 (4.8)	0.39±0.03	0 (0)	0	0.26
CTLD1-11	0 (0)	0	0 (0)	0	0.20
CTLD2-1	1 (2.4)	0.53	1 (3.1)	0.43	0.31
CTLD2-2	1 (2.4)	0.33	0 (0)	0	0.09
CTLD2-3	2 (4.8)	0.14±0.02	0 (0)	0	0.01
CTLD2-4-1	2 (4.8)	0.36±0.12	0 (0)	0	0.14
CTLD2-4-2	0 (0)	0	0 (0)	0	0.09

CTLD2-5	0 (0)	0	0 (0)	0	0.12
CTLD2-6	0 (0)	0	2 (6.3)	0.41±0.13	0.21
CTLD2-7	1 (2.4)	0.39	1 (3.1)	0.31	0.20
CTLD2-8	1 (2.4)	0.76	0 (0)	0	0.25
CTLD2-9	0 (0)	0	0 (0)	0	0.45
CTLD2-10	0 (0)	0	0 (0)	0	0.27
CTLD2-11	1 (2.4)	1.02	0 (0)	0	0.66
CTLD3-1	0 (0)	0	1 (3.1)	0.90	0.32
CTLD3-2	1 (2.4)	0.36	0 (0)	0	0.19
CTLD3-3	1 (2.4)	0.70	1 (3.1)	0.48	0.33
CTLD3-4	1 (2.4)	0.25	0 (0)	0	0.13
CTLD3-5	3 (7.1)	0.52±0.22	1 (3.1)	0.43	0.28
CTLD3-6	0 (0)	0	0 (0)	0	0.27
CTLD3-7	1 (2.4)	0.30	1 (3.1)	0.30	0.09
CTLD3-8	0 (0)	0	0 (0)	0	0.28
CTLD3-9	1 (2.4)	0.81	1 (3.1)	0.89	0.70
CTLD3-10	0 (0)	0	1 (3.1)	1.15	0.64
CTLD3-11	1 (2.4)	1.69	0 (0)	0	1.36
CTLD3-12	0 (0)	0	1 (3.1)	1.04	0.33
CTLD4-1	0 (0)	0	0 (0)	0	0.66
CTLD4-2	1 (2.4)	0.43	1 (3.1)	0.54	0.28
CTLD4-3	0 (0)	0	1 (3.1)	2.55	0.20
CTLD4-4	0 (0)	0	0 (0)	0	0.13
CTLD4-5	0 (0)	0	0 (0)	0	0.07
CTLD4-6	1 (2.4)	0.56	0 (0)	0	0.42
CTLD4-7	0 (0)	0	7 (21.9)	1.28±0.43	0.58
CTLD4-8	0 (0)	0	0 (0)	0	0.17
CTLD4-9	0 (0)	0	0 (0)	0	0.10
CTLD4-10	1 (2.4)	1.36	1 (3.1)	0.50	0.28
CTLD4-11	0 (0)	0	3 (9.4)	0.80±0.13	0.60
CTLD4-12	0 (0)	0	0 (0)	0	0.47
CTLD5-1	1 (2.4)	1.74	0 (0)	0	0.71
CTLD5-2-1	0 (0)	0	0 (0)	0	0.26
CTLD5-2-2	0 (0)	0	0 (0)	0	0.10
CTLD5-3	1 (2.4)	0.49	0 (0)	0	0.27
CTLD5-4	0 (0)	0	1 (3.1)	0.28	0.14
CTLD5-5	2 (4.8)	0.65±0.07	2 (6.3)	0.70±0.50	0.47
CTLD5-6	0 (0)	0	6 (18.8)	0.58±0.29	0.24
CTLD5-7	0 (0)	0	0 (0)	0	0.30
CTLD5-8	0 (0)	0	0 (0)	0	0.08
CTLD5-9	4 (9.5)	0.42±0.05	4 (12.5)	0.43±0.33	0.28
CTLD5-10	0 (0)	0	0 (0)	0	0.25
CTLD5-11	0 (0)	0	1 (3.1)	1.39	0.16
CTLD6-1	0 (0)	0	0 (0)	0	0.72

CTLD6-2	0 (0)	0	1 (3.1)	0.50	0.21
CTLD6-3	0 (0)	0	0 (0)	0	0.02
CTLD6-4-1	0 (0)	0	0 (0)	0	0.08
CTLD6-4-2	0 (0)	0	0 (0)	0	0.30
CTLD6-5	1 (2.4)	0.51	0 (0)	0	0.37
CTLD6-6-1	0 (0)	0	1 (3.1)	1.96	1.34
CTLD6-6-2	1 (2.4)	0.39	1 (3.1)	0.28	0.10
CTLD6-7	1 (2.4)	0.36	2 (6.3)	0.31±0.47	0.17
CTLD6-8	1 (2.4)	0.36	0 (0)	0	0.20
CTLD6-9	1 (2.4)	0.31	0 (0)	0	0.05
CTLD6-10	0 (0)	0	0 (0)	0	0.35
CTLD6-11	0 (0)	0	0 (0)	0	0.29
CTLD6-12	0 (0)	0	5 (15.6)	0.66±0.18	0.43
CTLD6-13	2 (4.8)	0.28±0.09	0 (0)	0	0.10
**CTLD7-1**	**6 (14.3)**	**0.28±0.05**	**2 (6.3)**	**0.27±0.07**	**0.11**
**CTLD7-2**	**7 (16.7)**	**0.58±0.1**	**12 (37.5)**	**0.58±0.12**	**0.34**
CTLD7-3	3 (7.1)	0.31±0.03	3 (9.4)	0.31±0.12	0.20
CTLD7-4	4 (9.5)	0.76±0.40	0 (0)	0	0.27
CTLD7-5-1	1 (2.4)	0.52	0 (0)	0	0.16
CTLD7-5-2	1 (2.4)	0.55	2 (6.3)	0.34±0.21	0.06
CTLD7-6	1 (2.4)	0.39	0 (0)	0	0.14
CTLD7-7-1	1 (2.4)	0.31	0 (0)	0	0.03
CTLD7-7-2	2 (4.8)	0.36±0.02	1 (3.1)	0.39	0.21
CTLD7-8	3 (7.1)	0.21±0.03	1 (3.1)	0.22	0.10
CTLD7-9	1 (2.4)	0.40	0 (0)	0	0.20
CTLD7-10	0 (0)	0	0 (0)	0	0.15
CTLD7-11	1 (2.4)	0.51	1 (3.1)	0.65	0.39
CTLD8-1-1	0 (0)	0	2 (6.3)	1.18±0.17	0.95
CTLD8-1-2	1 (2.4)	0.67	0 (0)	0	0.37
CTLD8-2	1 (2.4)	0.35	0 (0)	0	0.17
CTLD8-3	0 (0)	0	1 (3.1)	0.65±0	0.19
CTLD8-4-1	0 (0)	0	0 (0)	0	0.17
CTLD8-4-2	1 (2.4)	0.32	0 (0)	0	0.16
CTLD8-5-2	0 (0)	0	0 (0)	0	1.44
CTLD8-6-2	1 (2.4)	0.38	0 (0)	0	0.21
CTLD8-7	0 (0)	0	0 (0)	0	0.28
CTLD8-8	0 (0)	0	0 (0)	0	0.38
CTLD8-9	0 (0)	0	1 (3.1)	0.80	0.65
CTLD8-10	1 (2.4)	1.00	0 (0)	0	0.38
CTLD8-11	0 (0)	0	3 (9.4)	0.50±0.05	0.34
CTLD8-12	0 (0)	0	0 (0)	0	0.51
